# Genetic Population Structure of the Ground Beetle, *Pterostichus oblongopunctatus*, Inhabiting a Fragmented and Polluted Landscape: Evidence for Sex-Biased Dispersal

**DOI:** 10.1673/031.010.10501

**Published:** 2010-07-13

**Authors:** Malgorzata Lagisz, Kirsten Wolff, Roy A Sanderson, Ryszard Laskowski

**Affiliations:** ^1^ Institute of Environmental Sciences, Jagiellonian University, 31-202 Kraków, Poland; ^2^ School of Biology, University of Newcastle, Newcastle upon Tyne, NEI 7RU, UK

**Keywords:** gene flow, microsatellite markers, spatial structure

## Abstract

Ground beetles are an integral and functionally important part of many terrestrial ecosystems. Habitat change often influences population genetic structure of carabid beetles. In this study, genetic variation, population differentiation, and sex-specific dispersal patterns were studied in the forest ground beetle, *Pterostichus oblongopunctatus* F. (Coleoptera: Carabidae), in a fragmented and metal-polluted landscape to assess the consequences of human-induced changes on the population genetic structure. Genotypic variation at five microsatellite loci was screened in 309 beetles from 21 sample locations around zinc-and-lead smelter in southern Poland. Low levels of genetic differentiation among sampling sites were observed, suggesting high gene flow among populations. A negative correlation was found between levels of genetic differentiation and habitat patch size. No significant effects of metal pollution, in terms of genetic bottlenecks and genetic differentiation, were observed. Analyses revealed weak genetic clustering that is loosely tied to the geographic position of the sampled populations. Several tests of sex-biased dispersal were conducted. Most of them indicated male-biased dispersal. Differing levels of dispersal between females and males resulted in sex-specific spatial genetic patterns. Genetic differentiation was significantly correlated with geographical distance for males, but not for females, who were more diverged locally. Also, the effect of habitat patch size was sex-dependent, supporting the finding of different dispersal patterns between the sexes. This study demonstrated the application of microsatellite markers to answer questions regarding complex interactions between population structure and physical properties of the landscape. In the study system, migration appears to be sufficient to override potential effects of environmental pollution as well as habitat fragmentation. This investigation of population genetic structure indicated, for the first time, male-biased dispersal in carabid beetles.

## Introduction

Anthropogenic disturbance may drive species to extinction or to local adaptation. Extinction probability and micro-evolutionary processes are strongly linked to the organism's mobility, especially in fragmented landscapes (Thomas 2000; Carroll et al. 2007; Garant et al. 2007). Carabid beetles form a major family (Carabidae) of predacious and omnivorous species inhabiting a wide range of terrestrial habitats (Lindroth and Bangsholt 1985). They are widely used in biological surveys, especially to study effects of habitat alteration (Butterfield et al. 1995; Niemelä et al. 2002; Rainio and Niemelä 2003). Numerous studies have investigated the response of carabid species to changing environmental conditions resulting from human impact, such as forest fragmentation or management practices (Rainio and Niemelä 2002; Koivula 2000). Carabids are known to differ significantly in their dispersal abilities; flightless beetles are considered to be able to move a few hundred meters per day by walking, while species with good flight capability can move longer distances and are less dependent on dispersal corridors (Thiele 1977).

Obtaining accurate measures of dispersal in the field remains a problem when studying small species with obscure life styles, like most invertebrates. Direct observations using mark-recapture techniques are limited in geographic scope and time scale, and infrequent instances of long distance dispersal events can be easily missed (Jopp and Reuter 2005). Advances in molecular biology provide an opportunity to estimate short- and long-distance dispersal patterns in such species. Moreover, genetic methods allow for the determination of population structure, genetic variability and effects of habitat subdivision. Mobility of some of the species has been successfully investigated with molecular tools. For example, Keller and Largiader (2003) showed that gene flow and genetic variability in flightless *Carabus violaceus* was affected by the presence of major roads in the study area. Brouat et al. (2003), working on two other *Carabus* species, found that the forest specialist was more affected by habitat fragmentation than the forest generalist and that non-forested areas are only partial barriers to gene flow for both species. Several other papers also focused on effects of habitat isolation and heterogeneity on population differentiation and genetic diversity in carabids (Niehues et al. 1996; Desender and Serrano 1999; Desender and Verdyck 2001; Desender et al. 2005).

Dispersal rates can differ between the sexes. Although molecular methods are increasingly used to study sex-biased dispersal (Mossman and Waser 1999; Goudet et al. 2002; Prugnolle and de Meeus 2002), there is still little known about sex-biased dispersal in taxa other then vertebrates. Unfortunately, in most papers on population structure of terrestrial invertebrates, authors do not report whether they tested for differences in dispersal between the sexes. There are several studies where sex-biased dispersal has been found in insects including damselflies (Beirinckx et al. 2006), a few species of *Drosophila* (reviewed in Markow and Castrezana 2000), the mayfly *Callibaetis ferrugineus hageni* (Caudill 2003), the ant *Formica exsecta* (Sundström et al. 2003), and the bark beetle *Ips typographicus* ( Sallé et al. 2007).

The evolution of sex-biased dispersal is influenced by several factors. These include mating system, sex ratio, costs of dispersal, local competition for mates and resources, inbreeding avoidance, habitat persistence and dispersal timing (Gandon 1999; Perrin and Mazalov 2000; Hirota 2004). Most theoretical studies agree, however, that in polygynous or promiscuous species, males are predicted to be the more dispersive sex. In fact, dispersal tends to be male-biased in mammals (mostly polygynous) and female-biased in birds (mostly monogamous) (Greenwood 1980).

There are important evolutionary consequences to asymmetric dispersal rates. One is that a species may show different patterns of population structure for males and females (Prugnolle and de Meeus 2002). Theoretical models also predict that sex-biased gene flow may affect adaptive evolution in marginal sink environments (Kawecki 2003). Such environments are often of anthropogenic origin and can exhibit strong selective pressure leading to decrease in genetic diversity and/or genetic adaptation (Hebert and Luiker 1996).

This paper examines population genetic structure of the forest carabid, *Pterostichus* oblongopunctatus F. (Coleoptera: Carabidae), from fragmented and polluted habitats in southern Poland. Molecular data were used to test several hypotheses. Firstly, overall high dispersal rate of the species was expected, because the animals are relatively small and macropterous, thus potentially can disperse by flight. High migration rates are usually associated with low levels of genetic population structuring. Bottlenecks, founder effects, and evolution by drift were not likely to occur. Secondly, because the species can be reasonably expected to be promiscuous or polygynous, male-biased migration is expected. In genetic terms, it can be expressed as a higher proportion of shared alleles between sampling sites in males than in females, due to more frequent migration events in males. Finally, if overall dispersal rate is high, effects of habitat fragmentation and pollution on population genetic structure can be expected to be weak or absent. That means that there should be no correlation between genetic diversity and patch sizes or contamination levels. Response to habitat fragmentation and pollution can also be sex-dependent, with the more mobile sex showing greater genetic homogeneity over the study area.

## Materials and Methods

### Study species

*P. oblongopunctatus* is common to woodlands in the Palearctic region, occurring in both deciduous and coniferous forests (Lindroth and Bangsholt 1986). It is a generalist predator and a spring breeder; its predatory larvae grow during the summer in soil and litter (Müller and Kaschuba 1986). The generation time is generally one year, although a small proportion of adults may live up to three years (Brunsting 1981). Although *P. oblongopunctatus* is sometimes described as a non-flying species (Brunsting 1981), there is some evidence that a small proportion of individuals can disperse in this way (Van Huizen 1980; van Schaick Zillesen and Brunsting 1984). Males can be distinguished from females by the presence of the dilated anterior tarsal segments of the first pair of legs. The species is abundant in the study area, both at the polluted and non-polluted sites (personal observation). *P. oblongopunctatus* has been extensively used in ecotoxicological research in Poland, showing that environmental contamination has an impact on the species' life-history and physiology (Stone et al. 2001; Lagisz et al. 2002; Stone et al. 2002; Migula et al. 2004; Lagisz et al. 2005; Lagisz and Laskowski 2008).

### Sample collection

To assess the geographic distribution of genetic variation within and among populations of *P. oblongopunctatus*, samples were collected from 21 forest localities in Southern Poland (approximately 50° 15′ N 19° 25′ E to 50° 20′ N 19° 41′ E). All sampling sites were dominated by Scots pine, *Pinus sylvestris* L. (Pinales: Pinaceae) forest with a small number of other tree species, including oak, birch and rose. The sites chosen represent a broad range of metal pollution, with zinc, cadmium, and lead being the most important pollutants. The contamination levels at the sampling sites are reported as zinc concentration in the soil humus layer (Table 1) because this metal reaches the highest concentration, and the concentration and bioavailability of major metal pollutants in the region appears to be strongly intercorrelated (Zygmunt et al. 2006). Four soil humus samples from each site were taken during trapping of the ground beetles.

**Table 1.**
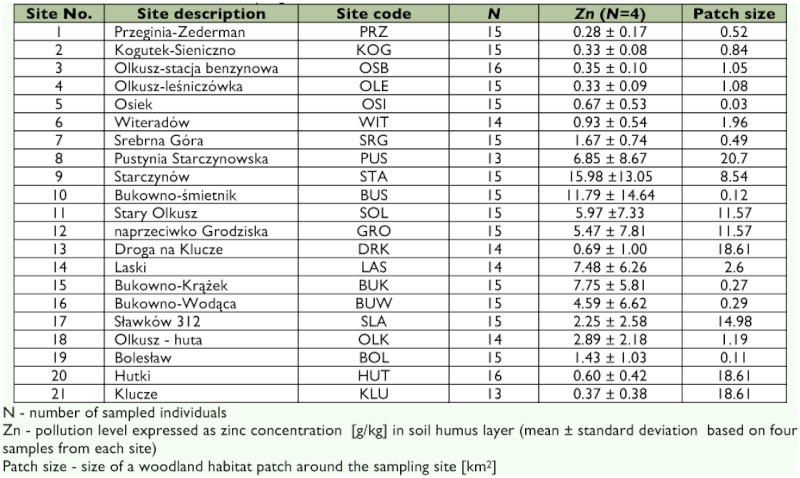
Characterization of the sampling sites.

**Figure 1.**
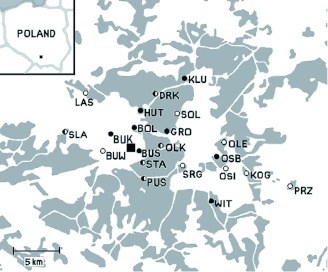
Study area. Circles — sampling sites, black square — smelter site, grey fields — woods. Circle filling colours show membership in genetic clusters according to Bayesian clustering method: Black and white-filled circles represent two main clusters with individuals' membership of at least 60%. Half-filled circles represent “unclustered” sites, comprised of individuals from two clusters in almost equal proportions (40–60% ancestry for each of the two clusters). High quality figures are available online.

Concentrations of Zn were analyzed with flame atomic absorption spectrometry (Perkin-Elmer AAnalyst 800, www.perkinelmer.com). Ten sampling sites showing extremely high levels of metal accumulation were located in close vicinity of the zinc-and-lead miningmetallurgic complex in Bolesław near Olkusz (Table 1). The remaining sampling sites were spread over areas with medium and low metal pollution. The geographic location of the sites is indicated in Figure 1. The region has a long history of mining and metal smelting. The peak levels of emissions (more than 1000 tonnes of dust per year) were noted in mid-20^th^ century when a big smelter was constructed. Because of falling production levels and cleaner production technologies, the emission of heavy metals from the local smelters decreased during the last 2 decades of the 20^th^ century to less than 50 tonnes per year (Stone et al. 2001). Carabid beetles used in the study were caught using pitfall traps during April– June 2002. From each site, 13–16 individuals (average 15), both males and females, were collected. Beetles were preserved in 99% ethanol until DNA extraction.

### Microsatellite typing

Genomic DNA was extracted from three legs from each individual using Chelex® 100 (Bio-Rad Laboratories, www.biorad.com). Tissues were dried at room temperature, ground in a 1.5 ml Eppendorf tube with a small pestle, and incubated in 500 µl 10% Chelex and 5 µl proteinase K (20 mg/ml) at 55° C for 3 h. Five microsatellite loci (Pob1, Pob3, Pob4, Pob5, and Pob14) were analyzed using primers and methods described by Lagisz and Wolff (2004). Amplification was performed in two multiplex PCR reactions: Multiplex 1 (0.2 µM of each of the forward and reverse primers: Pob1, Pob3, Pob4) and Multiplex 2 (primers Pob5 and Pob14). A total of 309 individuals (162 females and 147 males) from 21 sampling sites were used for assessing nuclear genetic variation. The microsatellite data was checked for scoring errors and null alleles with Micro-Checker v. 2.2.0 software (Van Oosterhout et al. 2004).

### Genetic diversity and gene flow between populations

For statistical analyses, beetles collected from each sampling site were assumed to represent local populations. Allele frequencies, observed heterozygosities, and unbiased estimates of expected heterozygosities (Nei 1978) were calculated with the SPAGEDI 1.1 package (Hardy and Vekemans 2002). Departures from Hardy-Weinberg equilibrium, linkage disequilibrium between loci, analysis of molecular variance,, population pairwise genetic distances, and migration rates were estimated with the software package ARLEQUIN 1.1 (Schneider et al. 1997). AMOVA was calculated over all populations to estimate intra- and inter-population variation (Excoffier et al. 1992; Weir and Cockerham 1984), without taking the geographic position into account. The *FST* fixation index (Weir and Cockerham 1984) was used because it is considered to be the appropriate statistic for small sample sizes and in cases when allele distributions show deviations from stepwise mutation model (Neigel 2002), which was observed in the investigated loci. Significance of fixation indices was tested using a nonparametric permutation approach with 1023 permutations (Excoffier et al. 1992).

To determine if there had been past bottlenecks in population size or founder effects at any locality, the BOTTLENECK v.1.2.02 program was used (Cornuet and Luikart 1996). This program tests if a significantly high number of loci show heterozygosity excess or deficiency relative to the expected heterozygosity computed under the equilibrium hypothesis from the number of alleles. A Wilcoxon signed rank test was chosen for this analysis, as this test does not require a large number of polymorphic loci. For this analysis, the two-phase model of microsatellite mutations was assumed (Cornuet and Luikart 1996).

Degree of genetic similarity among populations was estimated from an unrooted neighbor-joining clustering analysis of Cavalli-Sforza and Edwards's (1967) chord distance using POPULATIONS v.1.2.14 (Langella 2004). Confidence in tree topology was assessed by bootstrapping over loci (10,000 iterations) and the phylogenetic tree was visualized in TREEVIEW 1.6.6 (Page 1996).

To test whether the observed differentiation pattern could be better explained by a pure drift model or a model of equilibrium between gene flow and drift models, 2MOD software was used (Ciofi et al. 1999). In the pure drift model it is assumed that an ancestral panmictic population separated into several units diverging independently in complete isolation. The gene-flow model assumes that the gene frequencies within subpopulations are determined by a balance between genetic drift and immigration. The program also estimates *F*, the probability that two genes share a common ancestor within a population. A Markov Chain Monte Carlo simulation with 100,000 iterations was computed, and the first 10% of the output was discarded in order to avoid bias resulting from the starting conditions. Two independent runs were carried out to check the convergence of the posterior probabilities of the models. *F*-values were checked for convergence by comparing the means and time-series standard errors for the two runs. The number of migrants per generation (M) was estimated as (1 - *F*)/(4F) (Ciofi et al. 1999).

### Sex-biased dispersal

Sex-biased dispersal was investigated using BIASDISP 1.01 (Goudet et al. 2002), where four statistics are calculated and compared between the sexes: mean and variance of assignment index (*mAI_c_* and *vAI_c_*), *F_IS_* and *F_ST_*. The assignment index *(AI_c_)* determines the probability that a genotype originated from the population in which it is sampled. Most resident individuals are expected to have similar genotypes, and thus are more likely than immigrants to be assigned to their own population and therefore have higher *AI_c_* values. Consequently, the sex with a lower mean *AI_c_* has more potential recent immigrants. At the same time the more dispersive sex will have higher variance of assignment indices, because it will consist both of residents and immigrants (Goudet et al. 2002). For the same reason heterozygote deficit and resulting higher *F_IS_* values are expected in members of the more mobile sex, because *F_IS_* is a measure of how well the genotype frequencies within the population match Hardy-Weinberg expectations. A sample representing a mixture of resident and immigrant animals will exhibit heterozygote deficiency, and thus have a positive *F_IS_*. In contrast, *F_ST_* represents the proportion of the total genetic variance that attributed to among-population differentiation (Hartl and Clark 1997). The more dispersive sex carries new alleles to different populations, homogenizing them genetically. As result, lower values of *F_ST_* are expected for the more dispersive sex (Goudet et al. 2002). Significance testing for differences between the sexes in all statistics was based on 1000 randomizations.

### Environmental correlations: geographic distance, pollution, and fragmentation

Forest patch sizes and pairwise geographic distances between sampling sites were calculated using GIS software (Geographic Resources Analysis Support System GRASS, 1999–2002 GRASS Development Team). Forest patch sizes ranged from 0.03 to 20.7 km^2^ (Table 1). The smallest distance between any two sites was 1 km, and the largest was 21 km. The geographic distances between the pairs of sampling sites were log-transformed prior to the analysis. Pairwise genetic distances between the sampling sites were expressed as *F_ST_ /* (1 *F_ST_*), according to the method described by Rousset (1997).

Isolation-by-distance indicates the positive relationship between geographical and genetic (*F_ST_*) distance among populations. Counter-intuitively, its presence means that populations are inter-connected by gene flow and that the level of isolation is proportional to geographical distance. Isolation-by-distance was investigated using spatial autocorrelation analysis with SPAGEDI v.1.1 (Hardy and Vekemans 2002) by computing Moran's *I* statistics for 5 geographic distance classes between sampling sites (3.6, 5.6, 7.8, 10.2, and 20.1 km). The significance of each Moran's *I* (Hardy and Vekemans 1999) was calculated using a randomization procedure with 1000 permutations. Additionally, a Mantel test was performed to assess the impact of geographical distance on the amount of genetic differentiation between populations using the ‘Mantelise it!’ module of the FSTAT software package v. 2.9.3 (updated from Goudet (1995)), with significance tests performed over 10,000 randomizations.

To quantify the effects of habitat fragmentation and environmental pollution on genetic diversity a partial Mantel test was applied for all samples and for females and males separately (Smouse et al. 1986). Partial Mantel tests are typically used to calculate the partial correlation between two (or more) matrices, controlling for a third. Thus, this technique was used to examine the relationships between genetic distances and differences in pollution level and patch size, partialling out the effects of space. Additionally, the test provided information on the effects of spatial distribution of the sampling sites on observed genetic diversity over all samples and for females and males separately. For the calculations of partial Mantel tests the same software as for standard Mantel test was used (FSTAT).

### Population clustering analyses

Population heterogeneity was also assessed by using the program STRUCTURE v. 2.2 (Pritchard et al. 2000), which implements a Bayesian clustering method. Simulations were done with 100,000 burn-in length and 100,000 Markow Chain Monte Carlo replicates using no prior information and assuming correlated allele frequencies and admixture. Number of clusters (K) was calculated from 3–5 independent runs for each K value. The optimum number of clusters was identified using the second order rate of change of the likelihood function with respect to *K* (*ΔK*) (Chapuis and Estoup 2007). Sampling sites were placed into clusters based upon the highest percentage of assigned individuals' memberships. Due to generally high levels of admixture, a threshold value of 60% was used when assigning membership of the sampling sites in the determined clusters. It indicates that at least 60% of ancestry within a given site can be attributed to the respective cluster. The assigned sites were plotted on a map of the study region to examine geographical congruence of the clusters. Computations were repeated for males and females independently, using the same parameters as described above to detect differences in the clustering pattern.

Clusters determined for the total population were compared for differences in mean number of alleles, observed heterozygosity, gene diversity, *F_ST_* and *F_IS_*, using FSTAT software (15,000 permutations of the sites and two-sided test of the null hypothesis of no difference). The average pollution levels and patch sizes were compared between the clusters with ANOVA to determine if these environmental variables may be reflected in the clustering pattern. Finally, the samples were pooled within each cluster, and tests for sex-biased dispersal were repeated in the same way as described earlier.

**Table 2.**
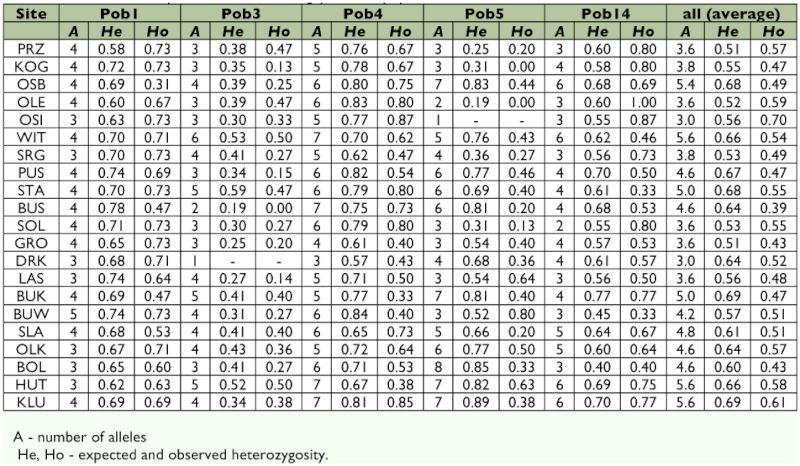
Genetic diversity measures of *P. oblongopunctatus* populations at 5 microsatellite loci.

## Results

### Genetic diversity and gene flow between populations

All five microsatellite loci investigated were polymorphic within and among populations with the exception of locus Pob3 in the population DRK and Pob5 in the population OSI (the three-letter codes refer to sampling sites, see Figure 1 and Table 1). The number of alleles, averaged over all loci, ranged from 3.0 in OSI to 5.6 in sites WIT, HUT and KLU. Private alleles were found at each locus, except Pob14, and generally occurred at low frequencies. Numbers of alleles and observed and unbiased expected heterozygosity values are given separately for each sampling site and locus in Table 2. Because the number of loci used in this study was small, and sample size from each location was limited, some caution was necessary in the interpretation of the results. Therefore, multiple approaches were used to confirm the robustness of the main findings.

For the linkage disequilibrium test, only 3 pairs of loci out of 210 pairs gave a significant p-value at the p < 0.0001 level. Single-locus tests for deviations from Hardy-Weinberg equilibrium revealed significant departure (at p < 0.0005) in 8 out of 105 analyses: 2 cases for locus Pob4 and 6 for locus Pob5. Hardy-Weinberg deviations were not restricted to particular sampling sites. In all 8 cases, the observed heterozygosity was lower than expected, which was probably not caused by null alleles because no null homozygote genotypes were observed. There was a difference in genotype distribution between the sexes, especially in the most variable locus, Pob5, where observed heterozygosity levels were generally lower in males than in females (data not shown). Deviation from Hardy-Weinberg equilibrium may indicate significant immigration, drift, selection and/or non-random mating.

**Figure 2.**
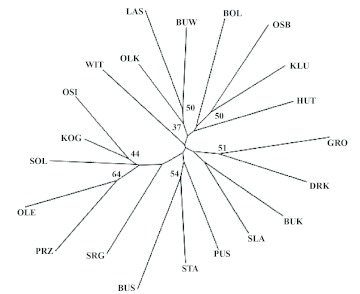
Unrooted neighbour-joining tree of 21 populations based on distances between populations estimated from Cavalli-Sforza and Edwards's chord distances. Phylogenetic trees were bootstrapped over loci (10,000 replicates). Numbers indicate percentage support of each branch in the topology; only the values > 30 are presented. High quality figures are available online.

The analysis of molecular variance (AMOVA) revealed statistically significant genetic structuring among the sampling sites: genetic variation within sampling sites amounted to 93.3%, whereas variation among the sampling sites was 6.7% (V_within_ = 1.40781, df_within_ = 597, V_among_ = 0.1011, df_among_ = 20, F = 3.1127, p < 0.0001). Locus by locus AMOVA showed that locus Pob5 is responsible for the largest part of the total genetic variation observed between the sites. For this locus, the variation among the sampling sites amounted to 21%. Loci Pob1, Pob4, and Pob14 also reflected significant differentiation, but to a smaller extent (2, 2.9 and 2.4%, respectively). Population pairwise estimates of *F_ST_* were less than zero in 12 cases, which were set to 0.0. The mean pairwise values for all sampling sites varied between 0.02 and 0.10.

To assess whether the allelic distributions within the sampling sites had been shifted by changes in population sizes due to the past population decline or colonization events, the BOTTLENECK program was used (Cornuet and Luikart 1996). Based on the two-phase model, no population exhibited significant heterozygosity excess or deficiency, indicative of recent bottlenecks (two-tailed Wilcoxon test run for each sampling site separately, all p > 0.05).

The population phenogram inferred from DCE distance illustrated very weak population grouping (Figure 2). The easternmost sites (PRZE, OLE, KOG, and OSI) are clustered together, and two neighboring heavily polluted sites, STA and BUS, form another group, with at least 50% bootstrap support. The way the other sampling sites tended to cluster seemed to be independent of their geographical origin. However, bootstrap values ranging from 6 to 64% suggest a low reliability of the observed clustering pattern.

**Table 3.**
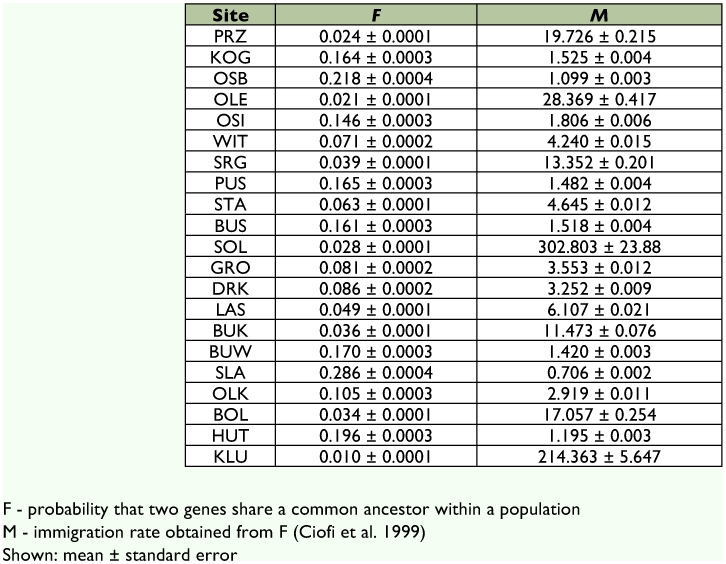
The relative interaction between gene flow and drift in sampled populations of the ground beetle *P. oblongopunctatus.*

Coalescent-based Markov Chain Monte Carlo method implemented in 2MOD (Ciofi et al. 1999) was used to test whether a pure genetic drift or a drift and immigration balance model would better explain the data. The results indicated that the gene flow model fitted the data best. The likelihoods of the gene-flow model and pure drift models were 1 and 0, respectively, i.e. none of the simulations supported the drift model.

Therefore, weak genetic differentiation of the *P. oblongopunctatus* populations results from a high level of gene flow between the sampling sites. Highest levels of immigration relative to drift were inferred in PRZ, OLE, SRG, SOL, BUK, BOL, and KLU with immigration rate *M* ranging from 11 to 302 individuals per generation (Table 3). Immigration values above 1 were found in all other sites, except the westernmost site SLA, where *M* was 0.706. This result shows that a large amount of migration between the sites was present, suggesting that the sampled populations of beetles were not isolated units.

### Sex-biased dispersal

In order to assess if there was sex-biased dispersal in the studied species, fixation indices over all loci were calculated separately for females (*n* = 162) and males (*n* = 147). The *F_ST_* values obtained were 0.082 for females and 0.066 for males. Although males appeared to be less genetically differentiated between populations than females, difference in *F_ST_* values between the sexes was not significant (permut sided, see hypotheses in the Introduction). The comparison of calculated *F_IS_* indices gave a significant difference between sexes (permutation test, p = 0.040), with males showing higher homozygosity levels (0.136 against 0.057 for females). Also, the assignment test showed that mean assignment index (*mAI_c_*) was low for males (-0.275) and high for females (0.250), and the difference between them ation test, p = 0.474, all tests one-was significant (permutation test, p = 0.036). At the same time, the variance among individuals in assignment indices (*vAI_c_*) was lower for females (4.90) than for males (6.36), but the difference was not significant (permutation test, p = 0.185). In summary, all calculated values that may reflect sex-biased dispersal supported a pattern that suggests a higher migration rate of males, although not all tests were statistically significant.

**Table 4.**
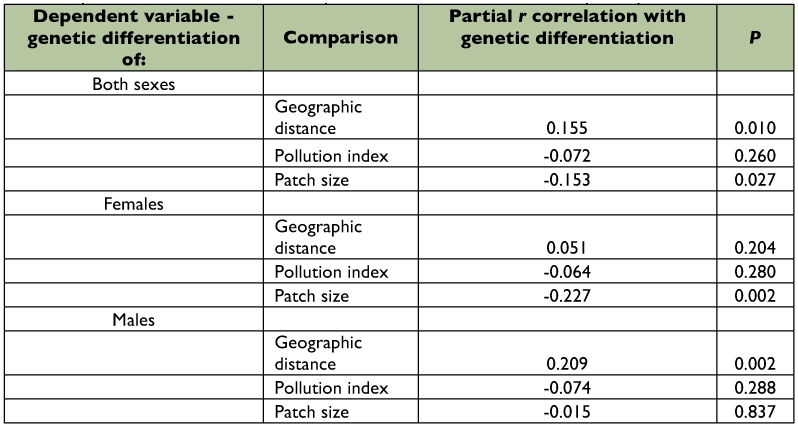
Partial Mantel r correlations between genetic differentiation (pairwise *F_ST_*) and geographic distance, pollution level and patch size, calculated for all samples, and for females and males, separately.

### Environmental correlations: geographic distance, pollution and fragmentation

**Figure 3.**
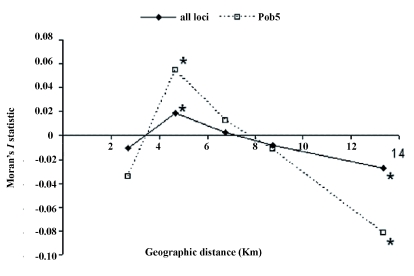
Spatial autocorrelogram estimated from multilocus microsatellite genotypes and for locus Pob5 only. * Moran's I significant at p 0.05. High quality figures are available online.

Spatial and genetic data were used to calculate Moran's *I* statistics, which expresses departures from spatial randomness. Moran's *I* values obtained in this study were significant and positive for distance classes 3.6 to 5.6 km (*I* = 0.018, p = 0.009) and negative for 10.2 to 21.0 km (*I* = - 0.027, *P* = 0.021, Fig. 3). Thus, genetic similarities among sampling sites appear to generally decrease with increasing geographical distance. Locus-by-locus tests showed that distribution of genetic variation in locus Pob5 was responsible for the observed relationship.

Similarly, significant isolation by distance was shown when pairwise *F_ST_* values and log-transformed geographic distance were correlated using a Mantel test (*r* = 0.155, p = 0.010). This result also reflects high migration rates between neighboring sampling sites. However, when data for females and males were analyzed separately with a Mantel test, correlation between geographic and genetic distance was significant for males (*r* = 0.209, p = 0.002) and insignificant for females (*r* = 0.051, p = 0.204).

Partial Mantel's tests were used to test for effects of habitat contamination levels and habitat patch size on distribution of genetic diversity, while controlling for potential effects of spatial autocorrelation. No significant associations were found between genetic differentiation and pollution over all individuals, as well as for females and males separately (Table 4). Negative correlation between levels of genetic differentiation and habitat patch size (*r* = -0.153, p = 0.028) was observed in partial Mantel tests calculated over all samples. However, when analyses were performed for two sexes separately, genetic diversity of females was related to forest patch size (*r* = -0.227, p = 0.002), whereas for males this relationship was insignificant (*r* = -0.015, p = 0.837) (Table 4).

### Population clustering analyses

The Bayesian analysis using STRUCTURE (Pritchard et al. 2000) indicated the presence of 2 main clusters in the total population. The highest value for *ΔK*, the rate of change in the log probability of the data between successive potential numbers of clusters (Evanno et al. 2005), was obtained for *K* = 2. Estimated log probability of the data was higher under *K* = 2 than under *K* = 1 (-3767 and -3891, respectively), therefore the two-cluster model was more likely than the single-cluster model. However, the revealed clustering pattern was weak, reflecting small genetic differentiation among the sampling sites. High proportions of admixed individuals were observed at all sites with assigned membership seldom exceeding 80%. Several sites showed approximately equal proportions of individuals assigned to different clusters, and they were thus classified as “unclustered.” The results were plotted on a map to evaluate the geographical relationships of the sites in different genetic clusters. The first cluster was composed mostly of relatively uncontaminated sites located in the eastern part of the study area. At the same time, five out of eight sites falling into the second cluster were grouped around the smelter site, generally more to the west than the first cluster (Figure 1).

The genetic variation, expressed as the mean allelic richness and gene diversity was significantly lower (p = 0.0004, permutation tests) in the eastern cluster (mean allelic richness = 3.49 and gene diversity = 0.49) than in the western cluster (mean allelic richness = 4.79 and gene diversity = 0.62) (Table 5). At the same time, mean observed heterozygosity was not significantly different between the two clusters (mean observed heterozygosity = 0.53 and 0.49, p = 0.265). Similarly, the level of differentiation among the sampling sites within the clusters was not significantly diverged between the clusters, although it tended to be higher in the eastern cluster (*F_ST_* = 0.73 and 0.32, respectively, p = 0.081). However, values of *F_IS_* were distinct (p = 0.0003), indicating dissimilarities in inbreeding levels between the clusters (*F_IS_* = -0.06 and 0.021). While sites in the western cluster were generally located closer to the smelter than those sites forming the eastern cluster, the difference in the mean zinc concentrations in the soil between the clusters was not statistically significant (ANOVA, *F* = 0.25, p_1,14_ = 0.624). The test for dissimilarities in mean patch size also gave a non-significant result (ANOVA, *F* = 1.79, p_1,14_ = 0.202).

**Table 5.**
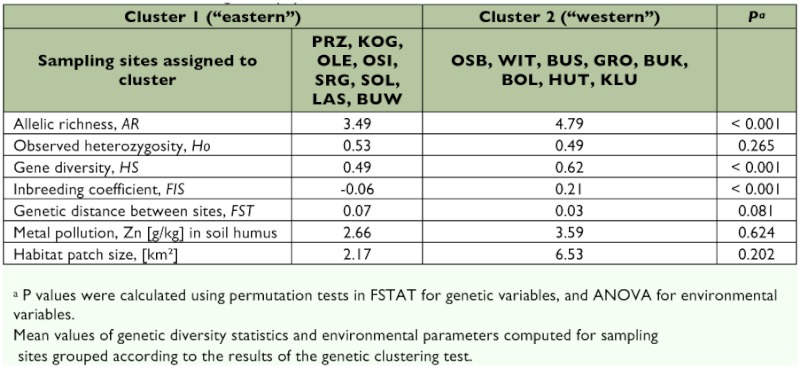
Characterization of the genetic population clusters.

The samples from different sites were pooled together by assignment to genetic clusters and re-analyzed for signatures of sex-biased dispersal. These tests revealed the same pattern, namely, that males are the more dispersive sex, as earlier tests on unpooled samples, when 21 sampling sites were treated as separate populations. The p-values were either higher or the same for all of the tests (*F_ST_*: p = 0.0855; *F_IS_*: p = 0.0295; *mAI_c_*: p = 0.0855; *vAI_c_*: p = 0.0855, permutation tests), reflecting increased test power. These results support the conclusion on the prevalence of male-biased dispersal.

## Discussion

### Genetic diversity and gene flow among populations

The data on genetic differentiation among the sampling sites provided support for the first hypothesis stated in the introductory section of this paper. Low levels of genetic population structuring were expected based on the relatively high mobility of macropterous ground beetles (Thiele 1977). Only 6.7% of the total genetic variation was observed between the sampling sites (AMOVA results). Nevertheless, the observed genetic differentiation was statistically significant, and most genotypic diversity was found within the populations. Such differentiation could potentially be caused by genetic drift or selection. Population divergence only by drift was excluded by the MOD2 analysis. Some impact of selection due to local differences in abiotic and/or biotic factors (eg. soil moisture or food availability) might still be likely as one locus is largely responsible for most between-population differentiation. It should also be kept in mind that observed deviations from Hardy-Weinberg equilibrium in allele distribution within populations may have caused overestimation of the level and significance of population differentiation (Chapuis and Estoup 2007). Signatures of recent bottlenecks were not detected, and no strong geographical clustering of populations was observed. Several analyses indicated that, although there was some significant genetic population structuring, populations of *P. oblongopunctatus* were linked by high levels of gene flow within and between habitat patches.

The lack of detailed ecological information on the organism's dispersal and gapcrossing ability made it difficult to assess and include in analyses the actual degree of geographic isolation experienced by *P. oblongopunctatus* populations. It is unclear what kind of habitat should be considered as hostile for this species and what the threshold distance is that separates two patches of suitable habitat.

This may have affected the results of this study, as some of the sampling sites assumed to be independent may function as one habitat patch, while others, considered as belonging to one woodland area should be treated as separated patches. Moreover, the presence of additional barriers, such as local roads and water bodies, may have had a significant influence on genetic differentiation of neighboring populations (Keller and Largiader 2003). Additional studies are needed to reveal if this is the case for *P. oblongopunctatus* and if the presence of such barriers explained the low Moran's *I* values observed in the first class of geographic distance (0–3.6 km, Figure 2).

### Sex-biased dispersal

Evidence was found for sex-specific patterns of population genetic structure. Allele frequencies observed in males were more similar among sampling sites than allele frequencies in females. Isolation-by-distance was evident for males, but not for females in the study area, indicating lower migration rates of females. These findings are supported by the results of a set of analyses for sex-biased dispersal based on several genetic parameters. It is possible that females usually move only for very short distances, i.e. mainly within habitat patches. In that case, isolation-by-distance could potentially be observed in females only at a much smaller geographic scale than used in the present study. However, based on available data, rare long-distance migration events cannot be ruled out for females, as such rare migrants are unlikely to be sampled from populations. While females appear to be the philopatric sex, males seem to disperse more often at the scale represented by our study, presumably searching for females during the reproductive period in early spring (Brunsting 1981). Males are also smaller and lighter, which may facilitate their dispersal by flight (Matalin 2003).

Dispersal mostly by one sex may not be sufficient to fully homogenise allele frequencies among subpopulations, so small differences in allele frequencies between the sexes can still prevail as observed in the present study (Goudet et al. 2002). That male and female beetles may not display the same dispersal abilities has implications for future research that was neglected in previous studies on carabid species. To our knowledge, this is the first such record for carabid beetles. This study shows that the difference in migration rates between the sexes can significantly affect population genetic structure of carabids.

### Environmental correlations: geographic distance, pollution, and fragmentation

The study revealed negative correlation between geographic distance and genetic differentiation, indicating higher migration rates between neighboring sampling sites than between more distant sites. However, this autocorrelation was statistically significant only for males and not for females. Significant isolation-by-distance detected for males indicated a higher migration rate for this sex. This result is in-line with the detected sex-biased dispersal. It is possible that landscape features, like geographical distance, habitat discontinuity, and patch size and/or quality, differently affect mobility of males and females. More dispersive males tend to homogenise the genetic structure of populations, regardless of patch size, while presumably more sedentary females tend to differentiate more among sampling locations, but still may have potential to occasionally colonise small and isolated habitatislets.

The results also demonstrated that levels of environmental pollution as encountered in the study sites have no detectable effect on population genetic structure of *P. oblongopunctatus.* It is known that pollutants may exercise directional selection on populations, but the effect of such a selection on population genetic structure in natural conditions is difficult to demonstrate (Van Der Wurff et al. 2003; Muller et al. 2007). Earlier research on *P. oblongopunctatus* showed that animals collected from polluted areas have reduced fecundity (Lagisz et al. 2002), tolerance to additional stressors (Stone et al. 2001), and altered enzyme activity (Migula et al. 2001; Stone et al. 2002) in comparison to beetles living in a non-polluted area. However, no substantial evidence of genetic adaptation has been found so far in *P. oblongopunctatus* (Lagisz et al. 2005; Lagisz and Laskowski 2008).

It is possible that contaminant stress did not induce a genetic change in exposed populations because the period of selection has been too short and/or the markers surveyed are not closely linked to genes involved in the development of resistance. The data presented shows no sign of significant genetic bottlenecks in these populations. Animals captured for this study from the most contaminated sites could represent either permanent local populations or recent immigrants. Thus, it is possible that lack of genetic differences between populations from contaminated and clean sites may simply result from intensive recolonisation of polluted sites after the levels of contaminants influx decreased during the last two decades. Significant gene flow between unpolluted and polluted areas is also likely to impair development of local genetic adaptation in the latter, thus preventing differentiation.

### Population clustering analyses

Population structuring pattern derived from Bayesian clustering analysis was more powerful than neighbour-joining clustering analysis and revealed two clusters of sampling sites. The general structuring was still weak, and the proportion of admixture was high at all sites, further supporting the prediction of a high dispersal rate of the species. The results of this study suggest the presence of a genetic cluster across the eastern part of the study area. The second cluster, containing populations located more to the west, and generally closer to the source of pollution is characterized by lower overall genetic diversity in comparison to the eastern cluster. Lower genetic divergence between the sampling sites and higher admixture levels within the sites forming the second cluster suggest higher dispersal rates in that area. However, there was no clear link between pollution level or habitat patch size and clustering pattern, which is also in-line with the results from the environmental correlations analyses.

Interestingly, when clustering was performed on the data from both sexes separately, the pattern observed in the females only was almost identical to that of the total data set. Isolation-by-distance, observed in males, but not in females, can make defining discrete genetic units problematic (Evanno et al. 2005) and therefore explains lack of clear clustering in males. The more dispersive sex is expected to present a larger heterozygote deficit and to be less genetically structured (Goudet et al. 2002). Finally, tests for biased dispersal on samples pooled accordingly to clustering pattern gave additional support to the conclusion of a male-biased dispersal.

### Conclusions

Our study illustrates how microsatellite markers can be used to reveal population genetic structure and sex-biased dispersal of a carabid beetle in a framework of an anthropogenically changed landscape. As expected, overall high dispersal rate of the species and low levels of genetic population structuring were detected. High gene flow among the sampling sites and genetic population clusters explains weak or no effects of habitat fragmentation and pollution on population genetic structure. Male-biased dispersal was demonstrated, as predicted from the species' biology.

## **Abbreviations**

**AMOVA**: analysis of molecular variance;
***F***: the probability that two genes share a common ancestor within a population;
*F_IS_*: inbreeding coefficient of an individual relative to subpopulation;
***F_ST_***: fixation index (the effect of subpopulations compared to total population);
***mAI_c_***: mean of genetic assignment index;
***vAI_c_***: variance of genetic assignment index
